# Magnetic Field Enhancement of an Electromechanical–Magnetic Antenna for ELF Cross-Medium Communication via a Parallel Configuration

**DOI:** 10.3390/s25206303

**Published:** 2025-10-11

**Authors:** Chung Ming Leung, He Chen, Menglong Liu

**Affiliations:** School of Robotics and Advanced Manufacture, Harbin Institute of Technology, Shenzhen 518055, China; cmleung@hit.edu.cn (C.M.L.); 24s153153@stu.hit.edu.cn (H.C.)

**Keywords:** ELF communication, cross-medium transmission, seawater communication, low-frequency wireless communication, electromechanical-magnetic antenna, magnetic field enhancement

## Abstract

Extremely low-frequency (ELF, 3–30 Hz) signals are effective for cross-medium transmission, yet conventional implementations are hindered by their large size and low efficiency. To address these limitations, a compact electromechanical–magnetic antenna (EMA) was developed and experimentally validated for ELF magnetic communication. The basic unit of the antenna, a single-EMA, consists of a stacked magnetostrictive composite beam, piezoelectric ceramic plates, and tip-mounted permanent magnets. The total envelope volume of a single EMA is only 3.3 cm^3^ with a maximum length of 12 cm, representing a substantial reduction compared with conventional ELF antennas. Building on this compact architecture, two EMAs were operated in parallel to form a parallel-EMA system, which significantly enhanced magnetic radiation through constructive magnetic coupling. Moreover, the optimal separation distance between the two EMAs was identified, ensuring efficient cooperative radiation. When driven at 50.2 mW, the parallel-EMA configuration generated a magnetic flux density of 114 pT at a transmission distance of 20 m in seawater. This performance demonstrates nearly a twofold improvement over a single-EMA unit, validating the scalability of parallel operation for stronger magnetic radiation. The compact form factor of the single EMA combined with the enhanced radiation performance of the parallel-EMA system enables portable ELF magnetic communication across diverse cross-medium scenarios, including air-to-sea and underground-to-air links.

## 1. Introduction

Electromagnetic (EM) waves face serious transmission challenges in conductive media and across media boundaries. When the difference in dielectric properties is large, refraction and reflection become especially complex [1,2,3]. In seawater, with a conductivity of about 4 S/m, strong eddy-current absorption causes severe attenuation above 1 kHz, particularly over long distances [4]. By contrast, extremely low-frequency (ELF, 3–30 Hz) signals penetrate different media with little loss, making them suitable for underwater communication and subsurface exploration [5,6]. The drawback is that conventional ELF antennas are bulky and inefficient, which restricts their use in compact or portable systems [7,8]. These challenges have motivated research into alternative antenna concepts.

To reduce antenna size at low frequencies, Liu et al. filed a patent in 2009 that proposed a mechanical antenna using rotating permanent magnets to generate EM waves [9]. Since then, mechanical antennas have been recognized as a promising route for compact low-frequency signal generation [10,11,12]. Reported designs generally fall into three categories: rotating permanent-magnet type, electret type, and piezoelectric-driven type. In the rotating-magnet-based design, a motor drives a permanent magnet to generate a time-varying magnetic field. For example, in 2018, Burch et al. [13] used a power drill to rotate a permanent magnet, producing an alternating magnetic dipole. With 138 W of input power, they obtained 800 fT at 100 m, confirming the feasibility of ELF generation with rotating magnets. Later, Wang et al. [14] introduced phased control and permanent-magnet arrays, achieving a 23.5% increase in radiation distance at 75 Hz. This clearly shows that array-based strategies have already been explored to improve radiation efficiency in rotating-magnet antennas. However, such systems still require bulky control assemblies, and modulation demands large, rapid variations in rotation speed, which place heavy demands on the motor.

Although rotating-magnet antennas are smaller than conventional EM antennas, their size is still not practical for portable use. To overcome this, researchers proposed electret-based mechanical antennas [15]. In this design, a charged electret replaces the permanent magnet, reducing bulk, while external motion produces a time-varying electric field for radiation. In 2017, Madanayake et al. [16] showed that a motor-driven polarized dipole could generate ULF signals. In 2019, Wang et al. [17] used a high-speed motor to rotate a polarized electret disk, simplified as a two-point charge model. Simulations indicated that at 100 Hz, the system produced a 1 fT magnetic field at 32 m, suggesting the potential of electret-based antennas for long-range ELF communication. In 2022, Cui et al. [18] introduced a new electret material (FEP/THV) with higher charge density. Simulations showed that this antenna generated a 1 fT ELF signal at 71.4 m in seawater with less than 5 W of power, confirming its communication capability. Even so, electret-based antennas still radiate weakly and share drawbacks with rotating-magnet designs. They depend on mechanical drives (albeit with smaller motors), and flexible modulation remains difficult, placing high demands on motor performance.

Given the strict motor requirements of rotating-based and electret-based antennas, researchers have increasingly turned to piezoelectrically driven designs. These antennas utilize the piezoelectric effect to convert electrical energy into mechanical motion, which is subsequently transferred to magnetostrictive materials to generate magnetic fields and radiate electromagnetic (EM) waves, often realized through magnetoelectric (ME) composites. The concept of magnetoelectric composites was first introduced in 1972 [19], where the direct ME effect was exploited to convert magnetic stimuli into electrical signals. Later, Mindlin demonstrated that a quartz plate driven by piezoelectric excitation could radiate EM waves, marking the earliest realization of piezoelectric-driven antennas [20]. However, studies on EM radiation from such devices remained limited for decades and only regained attention in recent years. In 2017, Domann et al. [21] analyzed their operating mechanism and confirmed the feasibility of EM radiation through piezoelectric or piezomagnetic vibration. That same year, Nan et al. [22] reported a miniaturized magnetoelectric antenna operating at 60.60 MHz, with dimensions one to two orders of magnitude smaller than conventional antennas at the same frequency. In 2019, Xu et al. [23] proposed a sandwich structure with a piezoelectric ceramic layer bonded between two magnetostrictive Metglas foils, offering a new compact design. Building on this, in 2020, Dong et al. [24] developed a piezoelectric-driven antenna using the sandwich structure, achieving very low-frequency (VLF, 3 kHz–30 kHz) communication at 23.95 kHz over 120 m, which demonstrated the potential of such designs for low-frequency operation. While piezoelectric-driven antennas eliminate the need for motors and improve miniaturization and efficiency, their operating frequencies are mainly limited by the resonance of piezoelectric and magnetostrictive materials, and most remain in the VLF rather than ELF band.

To further advance ME composites into the ELF regime, one notable structural innovation is exemplified by a Pb(Zr,Ti)O_3_-bimorph/NdFeB cantilever device, which exhibited a remarkably high ME response, reaching 250 V cm^−1^ Oe^−1^ (960 nC cm^−1^ Oe^−1^) at its fundamental bending resonance of ~60 Hz [25]. This approach offered a straightforward means of realizing strong ME coupling without the need for magnetostrictive layers. Subsequent studies on such configurations expanded rapidly. In 2015, Han et al. [26] demonstrated the harvesting of magnetic field energy at the power frequency of 50 Hz, reporting an output of 1.12 mW at 10 A and 9.40 mW at 40 A. More recently, in 2025, He et al. [27] developed an MME generator capable of capturing 50/60 Hz stray magnetic fields from power cables. Their design, based on PZT-5H ceramics, achieved a power density as high as 0.268 mW cm^−3^ Oe^−2^ and demonstrated excellent durability. Despite these advances, most low-frequency ME studies still emphasize sensing, leaving ELF radiation largely unexplored.

Here, we introduce a compact electromechanical-magnetic antenna (EMA) that achieves practical ELF radiation in a miniaturized form factor. The key innovations are: (i) a stepped-beam structure of equal width but varying lengths, with a thinner front arm that improves flexibility while reducing fracture risk under low-frequency drive [24]; (ii) a bimorph configuration of two piezoelectric plates, enhancing bending performance; and (iii) a parallel-coupled arrangement, where optimized spacing and polarity between units reinforce magnetic output without additional power. This integrated design breaks through the inherent trade-offs of rotating-magnet, electret, and piezoelectric-driven antennas, enabling ELF radiation with compactness and scalability. This integrated design breaks through the inherent trade-offs of conventional rotating-magnet, electret, and piezoelectric antennas. Since the operating frequency and physical volume of an antenna are closely related, to quantitatively benchmark miniaturization, we propose a new index—the volume-frequency density (VFD). The VFD is defined as:

(1) $$VFD=\frac{1}{f\times v}$$

where *f* is the operating frequency and *v* is the antenna volume. This index captures the fundamental trade-off between frequency and size: antennas designed for lower frequencies are generally expected to be larger. Detailed comparative results of VFD are summarized in Table 1. A larger VFD corresponds to a more compact design relative to its operating frequency. As shown in Table 1, the proposed EMA achieves the highest volume–frequency density (VFD) among reported mechanical antennas. Although piezoelectric-driven designs typically show only modest VFD values, our result is markedly higher. It should also be noted that in some rotating-magnet and electret systems, the motor volume was not included in the reported values; if fully considered, the advantage of our EMA would be even greater. In summary, rotating-magnet and electret antennas can operate at lower frequencies but are limited by bulky driving assemblies, while piezoelectric devices are compact but usually confined to the VLF band. By contrast, the present EMA uniquely combines compact size with true ELF operation, offering a promising pathway for next-generation portable ELF antennas.

## 2. Design and Working Mechanism of Electromechanical–Magnetic Antenna

**Figure 1:**
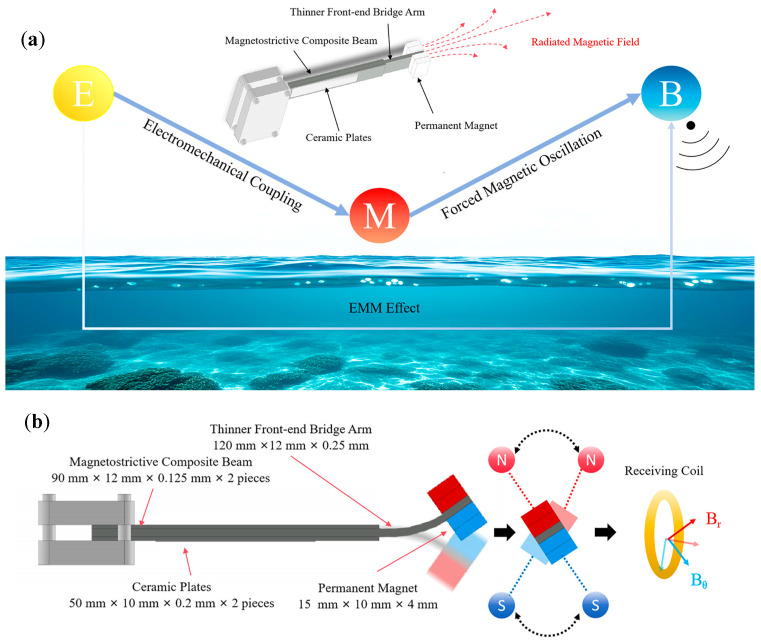
(**a**) Conceptual schematic of the proposed Electromechanical–Magnetic Antenna (EMA). The external electrical energy (E) is converted into mechanical energy (M) through electromechanical coupling, which in turn generates and radiates magnetic field energy (B). (**b**) Structural diagram and operating principle showing the stepped composite beam, ceramic plates, and tip magnet with the radiated field components ($B_{r}$, $B_{\theta }$).

The prototype of the proposed electromechanical–magnetic antenna (EMA) is shown in Figure 1. The antenna consists of three main components: a Metglas magnetostrictive beam, two piezoelectric plates, and four NdFeB permanent magnets. The beam was fabricated from Metglas magnetostrictive ribbons (Vacuumschmelze GmbH & Co., KG, Hanau, Germany) with a thickness of 25 μm, using an in-house hot-press method [23]. The beam comprised three layers: a central 120 × 12 × 0.25 mm^3^ magnetostrictive composite and two 90 × 12 × 0.125 mm^3^ magnetostrictive plates bonded to either side with high-strength epoxy resin (105/206, West System Inc., Bay City, MI, USA). This produced a single composite beam with uniform elastic properties, avoiding the mechanical joints or fasteners (e.g., screws) that often impair oscillation stability in heterogeneous assemblies. Two PZT-5H piezoelectric plates (Zhejiang Jiakang Electronics Co., Ltd., Jiaxing, Zhejiang, China), each 50 × 10 × 0.2 mm^3^ and poled in the same direction, were bonded to the rear section of the beam to form a bimorph structure, which was then integrated into a sandwich assembly. Electrical connections were made by attaching three wires: two to the outer surfaces of the PZT plates and one to the intermediate bonding layer. At the tip, four NdFeB permanent magnets were mounted to serve as a mass block. Each magnet had dimensions of 15 × 10 × 4 mm^3^ (≈600 mm^3^), corresponding to a volume of about 0.6 cm^3^. The overall antenna length was about 12 cm (excluding the mounting fixture). The device had an irregular geometry with an envelope smaller than 12 cm in length, 1.4 cm in width, and 1.25 cm in thickness. The maximum cross-sectional area was about 250 mm^2^, giving a total effective volume of about 3.3 cm^3^. Compared with conventional ELF antennas, this design represents a significant reduction in size.

In detailed operation, an applied voltage drives the piezoelectric plates through the converse piezoelectric effect, producing strain that is transferred to the Metglas beam and induces bending vibrations. The two PZT-5H plates are connected in a bimorph configuration with opposite poling, so that one expands while the other contracts, effectively doubling the bending moment compared with a unimorph design and enhancing tip displacement of the NdFeB magnets [34]. With the parallel-elastic design of the Metglas beam, the reduced stiffness at the tip allows larger motion of the magnets, while the tip mass further amplifies the oscillation, strengthening the radiated magnetic field. In this way, the oscillation of the tip magnets generates an alternating magnetic field. Compared with unimorph ELF antennas, the bimorph-based design achieves larger tip displacement while maintaining structural stability.

Although the bimorph introduces additional dynamic effects, the resulting field can still be approximated by a classical dipole model. The general expression of a dipole magnetic field follows the classical form [24]. In our design, however, the bimorph action effectively amplifies the oscillation angle of the tip magnets, and this newly incorporated factor is reflected in Equation (2). Under these conditions, the alternating magnetic field (B) generated by the proposed EMA can be expressed as:

(2) $$B=\frac{B_{r}V_{m}\beta }{4\pi r^{3}}\{2\cos\theta \dot{r}+\sin\theta \dot{\theta }\}$$

where *B*_r_ is the remanent magnetic flux density of the magnet, *V*_m_ is the volume of the NdFeB permanent magnet, *r* is the distance from the magnet to the observation point, and *θ* is the instantaneous tilt angle of the magnet. $\dot{r}$ and $\dot{\theta }$ represent the 90° horizontal component (along the magnetic dipole axis) and 0° vertical component (perpendicular to the axis) of the magnetic field at the receiving point, respectively. The alternating field is maximized when *θ* = 0, i.e., when the magnet oscillates along the *z*-axis.

**Figure 2:**
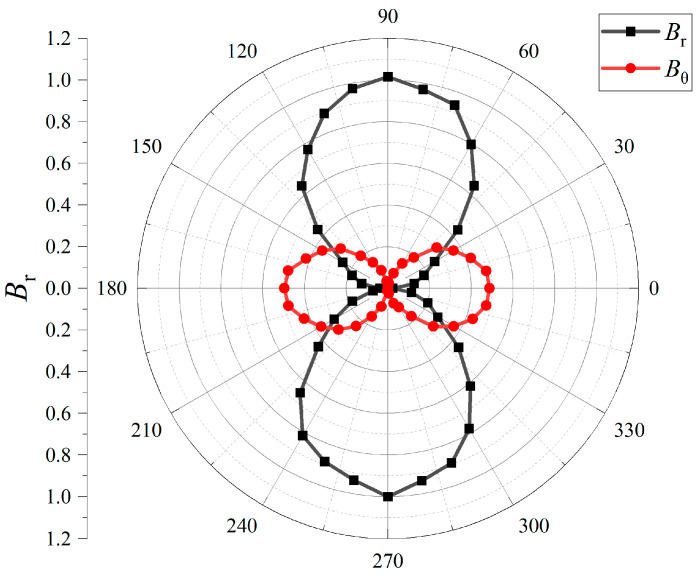
Measured near-field radiation patterns of the proposed EMA, showing the radial component ($B_{r}$) and tangential component ($B_{\theta }$).

The measured near-field radiation patterns of the EMA in Figure 2 show a dipole-like distribution consistent with Equation (2). The radial component ($B_{r}$) dominates along the axis, while the tangential component ($B_{\theta }$) is strongest in the transverse plane. This confirms that the tip magnet produces the expected alternating dipole field. The clear separation of *B*_r_ and *B*_θ_ also indicates stable oscillation without distortion, which is important for predictable coupling in communication scenarios. Small deviations from the ideal pattern are attributed to fabrication tolerances and measurement uncertainty, but the overall match with the model is preserved.

To further increase *B*, enlarging the oscillation amplitude of the tip magnet is essential. By classical thin-plate theory, the bending stiffness (*D*) of a plate is [35]:

(3) $$D=\frac{{Eh}^{3}}{12(1-\nu^{3})}$$

where *E* is Young’s modulus, *h* is the plate thickness, and ν is Poisson’s ratio. For a given material, *D* $\propto h^{3}$; thus reducing thickness decreases *D* and increases compliance under the same load. In our proposed EMA, this classical relationship is deliberately exploited: the front end of the beam is designed with magnetostrictive laminates of half the thickness of those at the rear. This intentional reduction in *D* near the tip increases the oscillation amplitude and the displacement of the tip-mounted permanent magnet, thereby enhancing the radiated magnetic field. This thickness-graded configuration represents a distinctive contribution of our work compared with conventional uniform-thickness beams.

## 3. Experimental Characterization of Magnetic Field Radiation in EMA

**Figure 3:**
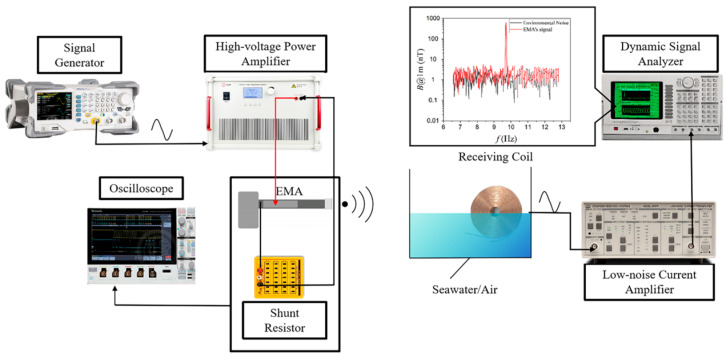
Measurement setup for the magnetic field radiation characteristics of the proposed electromechanical-magnetic antenna (EMA).

The experimental evaluation began with measurements of the magnetic field radiation from the proposed Electromechanical-Magnetic Antenna (EMA). The setup is shown in Figure 3. It consisted of a signal generator (RIGOL DG1022Z, RIGOL Technologies Co., Ltd., Beijing, China), a high-voltage power amplifier (Aigtek ATA4052, Aigtek Co., Ltd., Xi’an, China), a dynamic signal analyzer (SRS SR785, Stanford Research Systems, Inc., Sunnyvale, CA, USA), and a low-noise current preamplifier (SRS SR570, Stanford Research Systems, Inc., Sunnyvale, CA, USA). A 100 Ω shunt resistor was connected in series with the EMA to monitor input current. Because the operating frequency was extremely low, the induced signal was captured by a 3710-turn copper receiving coil, which was pre-calibrated in-house. The coil was placed in a 1.2 m × 40 cm water tank. For air measurements, the tank was empty, while for seawater tests, seawater was added and the coil was submerged. The output was then amplified and analyzed by the current amplifier and dynamic signal analyzer. This setup enabled detection of weak ELF signals with adequate sensitivity. To reduce environmental ELF noise, all possible interfering devices were switched off, and tests were carried out late at night when background disturbances were minimal. To monitor drive conditions and calculate input power, a digital oscilloscope was connected in parallel to the amplifier output and across the 100 Ω shunt resistor. The oscilloscope recorded the time-domain drive voltage (*v*_in_(*t*)) applied to the EMA and the shunt voltage (*v*_R_(*t*)). The instantaneous current was calculated from Ohm’s law as $i\left(t\right)=\frac{v_{R}\left(t\right)}{100\;\Omega }$, and the input power (*P*_in_) was calculated accordingly.

In practice, the receiving coil was treated as a calibrated sensor after pre-calibration. The SR570 provided low-noise gain and selectable filtering to suppress out-of-band disturbances, while the SR785 performed spectral estimation and narrow-band extraction at 9.7 Hz to reject mains hum and harmonics. Coaxial cabling and single-point grounding were used to minimize loop pickup. Repeated measurements and signal averaging were employed to improve the signal-to-noise ratio and ensure repeatability.

**Figure 4:**
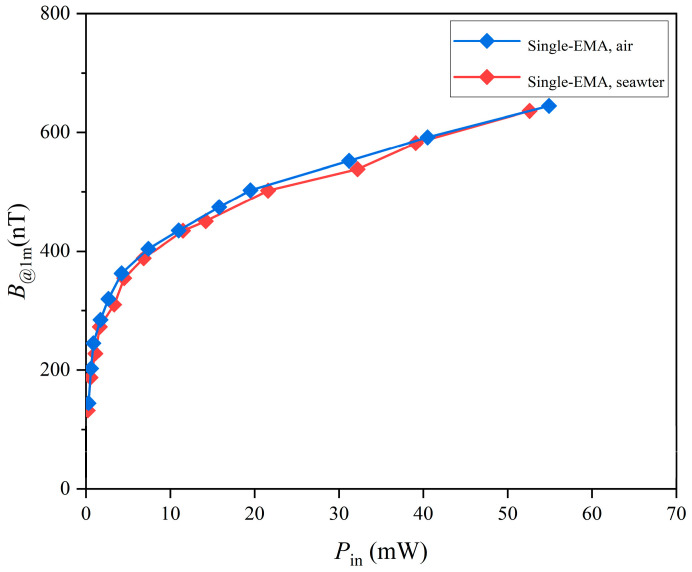
Magnetic flux density at 1 m versus input power for a single EMA, measured in air and in seawater (operating at 9.7 Hz).

To evaluate the transmission capability of ELF radiation, the magnetic flux density (*B*) at a distance of 1 m was measured as a function of input power (*P*_in_) in both air and seawater, as shown in Figure 4. seawater used in the experiments had salinity ≈ 35%, temperature ≈ 24 °C, density ≈ 1023.5 kg/m^3^, pH ≈ 8.0, and turbidity < 1 NTU. Measurements were carried out at 9.7 Hz, which was the resonance frequency of the EMA. The resulting *B*-*P*_in_ curves for air and seawater nearly overlapped, indicating that ELF signals propagate effectively in both media, with minimal attenuation due to seawater conductivity at this frequency.

When *P*_in_ was below 10 mW, the magnetic flux density increased rapidly with power, but the growth saturated at higher levels, indicating that increasing drive power does not indefinitely enhance EMA radiation. At 54.9 mW, the measured flux density reached 644.5 nT at 1 m in seawater. At 10 Hz, the background geomagnetic noise in air has been reported in the literature to be on the order of 200 pT [36]. In seawater, our measurement showed that the effective background noise increased, with an average value of 1.33 nT at 1 m [37]. Although our coil-based setup was not able to resolve the very low noise floor in air, the seawater measurement confirmed a clear signal-to-noise margin for reliable detection. These results suggest that the usable transmission range in seawater is ultimately limited by the higher background noise, even though ELF attenuation in seawater is intrinsically small.

To address the observed saturation of radiation strength at higher driving powers, a parallel configuration was adopted in which the input power was shared across multiple units. This arrangement increases the overall driving capacity of the system while reducing the load on individual actuators, keeping them within a stable operating range and avoiding instability under high-power conditions. In the proposed antenna, the radiated magnetic field is generated by the oscillatory motion of the front-end NdFeB permanent magnets. When multiple EMAs operate together, their magnetic output is determined by the coupled motion of the tip magnets through mutual interaction, which directly influences the overall radiation performance. The relative orientation of the magnets is therefore critical. In the NS-NS configuration, where the north pole of one magnet faces the south pole of the other, the fields are in phase and reinforce each other, producing constructive interference and stronger radiation. In contrast, in the NS-SN configuration, the fields are out of phase, leading to partial cancelation and reduced efficiency. For this reason, the NS-NS orientation was selected for further study.

**Figure 5:**
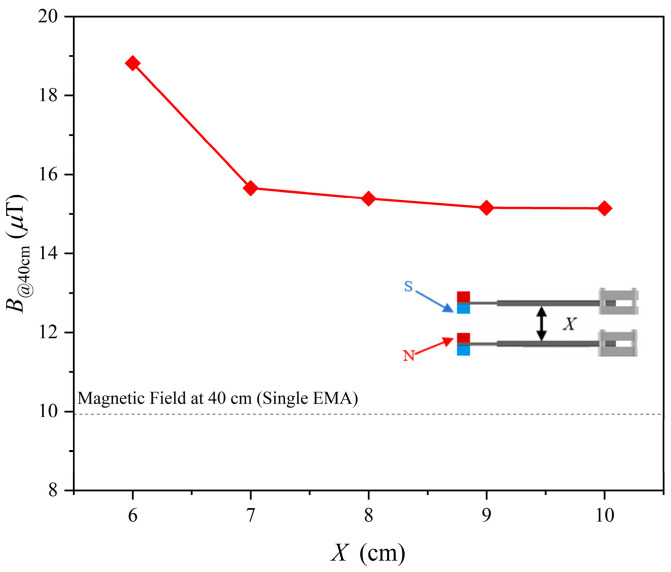
Magnetic field strength (*B*) at 40 cm as a function of parallel spacing (*X*) when the mutual-attraction configuration of two EMAs (parallel-EMA). The dash line indicates the reference field strength of a single EMA.

The effect of spacing in this configuration was evaluated by measuring the magnetic flux density at 40 cm $\left(B_{@40cm}\right)$ for different center-to-center distances (*X*). The results (Figure 5) show that $B_{@40cm}$ reaches a maximum at *X* = 6 cm compared with the single-EMA baseline. When *X* < 6 cm, the attractive force between magnets becomes excessive, drawing the beams together or biasing their motion, which can induce mechanical locking and prevent sustained oscillation. At larger separations, the coupling effect weakens: for *X* > 6 cm, the enhancement decreases noticeably, and beyond *X* = 6 cm, the measured gain becomes small. These results indicate a critical spacing range in which magnetic coupling strongly influences radiation. The measured enhancement was lower than the theoretical doubling of field strength (a factor of two, or 6.02 dB), likely due to fabrication tolerances and slight misalignments of the tip magnets. Additional measurements showed that a single EMA consumed 52.6 mW, while the parallel configuration consumed 76.5 mW, highlighting the importance of improved fabrication precision to fully realize the benefits of magnetic coupling.

To quantify the radiation efficiency improvement achieved by the parallel-EMA, the relative enhancement η was calculated as:

(4) $$\eta \;=\left(\frac{B_{in\;Parallel}}{B_{single}}\times \frac{P_{single}}{P_{in\;Parallel}}\right)\times 100\%-1$$

where $B_{in\;Parallel}$ is the magnetic flux density produced by the parallel-antenna system, *B*_single_ is that of a single antenna, *P*_single_ is the input power to the single antenna, and $P_{in\;Parallel}$ is the total input power to the parallel-antenna system.

**Figure 6:**
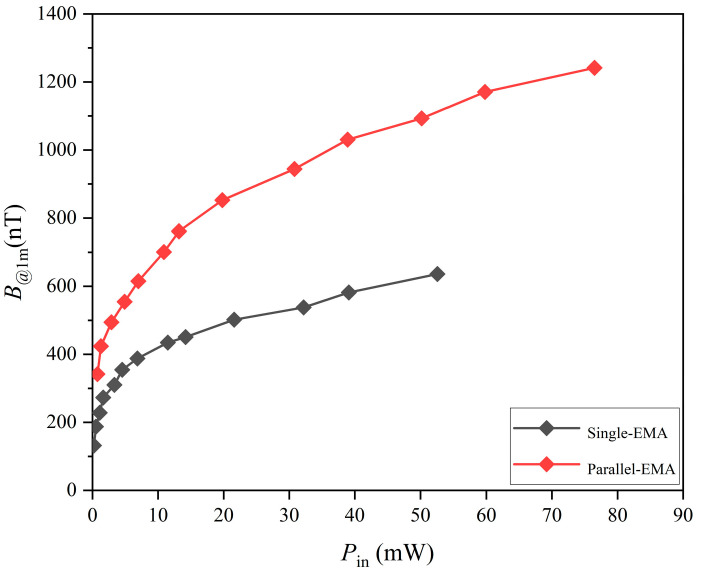
Comparison of the radiated magnetic flux density as a function of input power between single-EMA and parallel-EMA in seawater.

To further analyze the radiation efficiency of the parallel-EMA system, the comparison shown in Figure 6 was examined in greater detail. The curve corresponding to the parallel-EMA configuration clearly exhibits a steeper slope in the higher-power regime, indicating that the enhancement effect becomes most prominent at relatively elevated driving powers. This trend suggests that the magnetic coupling between the two EMAs is capable of compensating for the intrinsic radiation inefficiency that typically arises at higher excitation levels, thereby extending the usable dynamic range of the antenna system. At the same time, the single EMA shows a slower growth in magnetic flux density, reflecting the limitations of a solitary resonant structure in sustaining efficient energy conversion as the input power increases. As the driving power continues to rise, both curves gradually enter a saturation region. Nevertheless, the parallel configuration consistently outperforms the single EMA by a substantial margin, underscoring the robustness of magnetic coupling in maintaining superior field radiation efficiency. This behavior is particularly relevant for scenarios that demand stable ELF transmission under variable excitation conditions, where the parallel-EMA system can provide stronger and more reliable signals without requiring excessive increases in input power. From the quantitative measurements, the parallel-EMA system (at the optimal spacing of *X* = 6) produced a magnetic flux density (*B*) of 1170.8 nT at 1 m with an input power (*P*_in_) of only 59.8 mW. In comparison, a single antenna driven at 32.2 mW (approximately half the total input power) produced only 538.07 nT at the same distance. Substituting these values into Equation (3) gives η≈17%, confirming that the parallel configuration delivers a notable enhancement in magnetic field radiation efficiency.

Beyond efficiency, the parallel configuration also mitigates the saturation of EMA radiation strength at high input powers. By distributing the load across multiple units, the system avoids excessive stress on individual actuators and supports higher driving powers with stronger outputs. This directly addresses a limitation of current miniaturized ELF mechanical antennas, where radiation is restricted by power saturation. These results demonstrate that parallel EMA architectures offer a practical route to achieving stronger and more stable radiation in compact ELF systems.

## 4. Cross-Medium Transmission Experiment in Seawater

**Figure 7:**
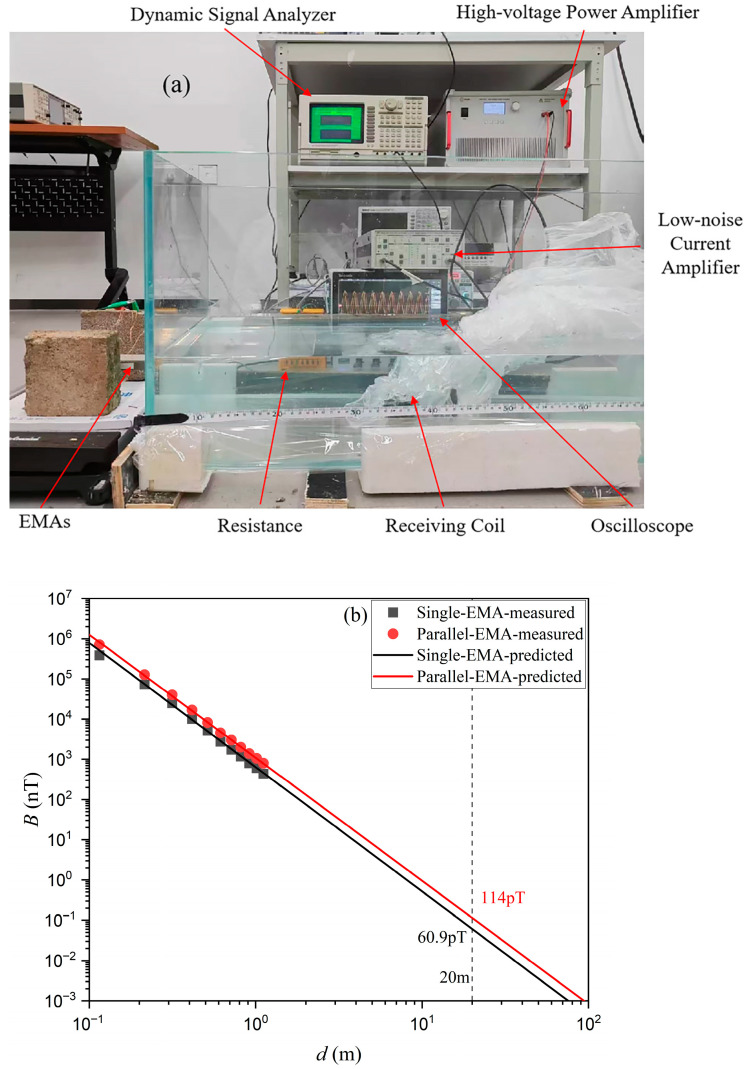
(**a**) Photograph of the experimental setup for the ELF magnetic field communication system across the air-seawater interface, showing the key instrumentation and components. (**b**) Comparison of measured and predicted magnetic flux density (*B*) as a function of transmission distance (*d*) for single-EMA and parallel-EMA in seawater.

To evaluate the practical performance of the proposed EMA, an indoor cross-medium transmission setup was built, as shown in Figure 7a. The seawater used in the tests had a conductivity of about 4 S/m. The receiving antenna was sealed inside a plastic enclosure and immersed in the seawater tank, while the transmitting EMA was placed in air. In this way, the radiated signal traveled across the air-seawater boundary before being detected. This arrangement replicated key features of realistic cross-medium transmission, where both conductive losses in seawater and impedance mismatch at the interface affect signal strength. All electronic instrumentation and connections were kept identical to those in the baseline air-only experiments, with the only change being the introduction of the seawater tank. This ensured that any performance differences could be attributed to the seawater medium rather than experimental variations.

Figure 7b shows the measured magnetic flux densities of the single EMA and the parallel EMA at different transmission distances (*d*). At 20 m, the single EMA produced 60.9 pT and the parallel EMA produced 114 pT. Measurements were taken up to 1.2 m, and longer ranges were extrapolated by fitting the data to a magnetic-dipole model in seawater (σ≈4 S/m) with the expected $\frac{1}{r^{3}}$ decay. The fitted curves match the measured data closely, supporting the validity of the extrapolated results. The twofold increase in *B* from the parallel EMA system results from constructive coupling between the two emitters, which increases the effective aperture and offsets the efficiency limits of a single EMA. The close agreement between measured and predicted values indicates that the simplified analytical model remains valid across the air-seawater interface, despite the additional conductive loss. From a system perspective, the 114 pT measured at 20 m with an input power of 50.2 mW corresponds to a meaningful extension of useful link range compared with a single EMA. While the received signals at such distances approach the noise floor of low-frequency receivers, the additional ~53 pT margin provided by the parallel configuration can reduce bit-error rates or shorten integration time during demodulation. This demonstrates that parallel EMA configurations can provide more robust cross-medium links in seawater, where conductive attenuation, boundary mismatch, and multipath effects often constrain performance.

Besides the VFD analysis, Table 1 also compares the proposed EMA with prior mechanical antennas in terms of drive power, magnetic flux density, and transmission distance. Rotating-magnet designs typically operate within 1–500 Hz but require hardware volumes exceeding 100 cm^3^. Even with measurable outputs—for example, 20 nT at 5 m [13]—their large size and reliance on moving parts restrict scalability and integration. Piezoelectric and electret approaches are more compact and usually operate at tens of kilohertz. They can generate strong local fields (e.g., 5.63 nT at 8 cm [18]) and have been demonstrated for far-field detection (e.g., 100 fT at 92 m [15]), but they rely on high carrier frequencies and are difficult to adapt to ELF operation. Their performance also declines in conductive environments, where attenuation increases at higher frequencies. Piezoelectrically driven antennas ([27,28]) are another option, but they generally work above 30 kHz, outside the ELF band that is critical for underwater and underground communication. In contrast, the proposed EMA operates at 9.7 Hz with an effective volume of ~6.6 cm^3^ and a drive power of ~50.2 mW, achieving 114 pT at 20 m. This corresponds to ELF radiation with a footprint several orders of magnitude smaller than rotating-magnet systems, while avoiding the high-frequency requirements of piezoelectric or electret resonators. The combination of ELF operation, compact form factor, and moderate power demand positions the EMA as a practical solution that bridges the gap between bulky low-frequency rotors and compact high-frequency resonators. These features make it well suited for embedded or mobile platforms where size, energy efficiency, and cross-medium penetration are critical.

**Figure 8:**
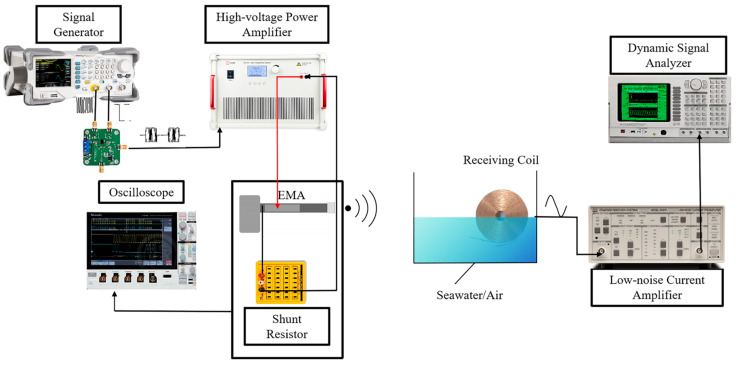
Schematic diagram of the experimental setup for ASK-based cross-medium communication.

Figure 8 shows the experimental setup used to demonstrate amplitude shift keying (ASK) modulation with the EMA. The goal was to examine modulation and demodulation across the air-seawater interface under controlled conditions. In this setup, the modulated drive excites the EMA, generating an ELF magnetic field that propagates through seawater and is captured by the receiving coil. The configuration is similar to the earlier measurement system but includes an additional multiplier to generate the ASK-modulated signal. Both channels of the signal generator were used to provide the carrier and baseband inputs for modulation. The received waveform was then amplified and analyzed to recover the ASK envelope. These results confirm that the EMA can work with digital modulation and demonstrate the feasibility of cross-medium ELF magnetic communication.

**Figure 9:**
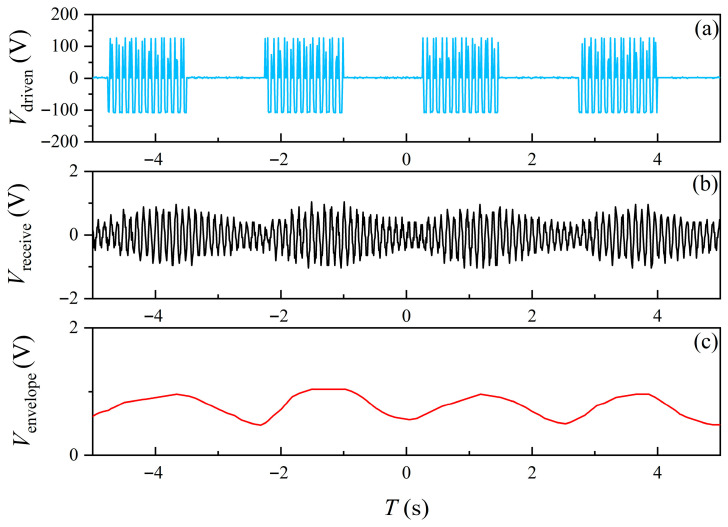
Experimental results of amplitude shift keying (ASK) transmission in seawater: (**a**) transmitted ASK-modulated voltage waveform, (**b**) received carrier signal, and (**c**) extracted envelope signal.

Figure 9 presents the experimental results of amplitude shift keying (ASK) modulation and demodulation in seawater. Figure 9a shows the applied driving voltage ($V_{driven}$) for the transmitting EMA, where the sinusoidal carrier is periodically switched on and off according to the digital sequence. The observed peak driving voltage was about ±150 V, sufficient to generate detectable magnetic flux density in seawater. The blank intervals represent the “0” symbols, while the sinusoidal bursts correspond to the “1” symbols. Figure 9b displays the signal received by the coil. Despite attenuation of electromagnetic waves in seawater, the waveform remains visible, and the amplitude of the received signal ($V_{receive}$) shows clear bursts corresponding to the driving sequence. This indicates successful transmission of modulated information across the seawater channel. Fluctuations in amplitude are also seen, likely due to multipath effects, ambient noise, and the frequency-dependent loss in seawater, which are typical challenges in underwater communication, especially at low frequencies. The received output was directly transferred to a PC, where envelope extraction was carried out through digital processing. Figure 9c shows the recovered signal envelope ($V_{envelope}$), which follows the amplitude variation in the transmitted ASK waveform, allowing reliable demodulation of the binary sequence. The distinction between active and inactive intervals confirms that ASK modulation is feasible for mechanical antenna communication in seawater. Compared with alternatives such as FSK or PSK, ASK offers a simpler structure and less demanding detection, making it attractive for low-power and resource-limited underwater applications.

## 5. Conclusions

In summary, this work presents a compact electromechanical–magnetic antenna (EMA) composed of a stepped magnetostrictive beam, a PZT-5H bimorph driver, and a tip-mounted NdFeB permanent magnet, with an effective volume of about 3.3 cm^3^ and a length of ~12 cm. The stepped design enables large tip displacement while maintaining structural stability, allowing efficient ELF magnetic radiation in a form factor suitable for portable use. Systematic tests confirmed the expected growth-saturation behavior of the radiated field with input power, showing that higher drive levels alone cannot indefinitely enhance output. To address this, a parallel-EMA arrangement was introduced, which distributes the drive and benefits from constructive magnetic coupling. With NS-NS orientation and optimized spacing, the parallel configuration consistently exceeded the performance of a single unit. Quantitative results showed a flux density of 1170.8 nT at 1 m with 59.8 mW input, with the efficiency improvement captured by a normalized enhancement metric. Cross-medium experiments further verified signal transfer across the air-seawater interface. Measurements up to 1.2 m, combined with dipole fitting, yielded range estimates of 60.9 pT (single-EMA) and 114 pT (parallel-EMA) at 20 m in seawater, demonstrating nearly twofold gain while remaining consistent with the channel model. Finally, ASK modulation was implemented as a proof of concept: the received signal showed clear symbol bursts and envelope detection produced reliable two-level outputs, indicating the compatibility of the EMA with low-complexity digital modulation at ELF in conductive media.

## Figures and Tables

**Table 1:** Comparison of reported mechanical antennas using the proposed volume-frequency density (VFD) as a performance metric.

Method	Ref.	Freq. [Hz]	Volume. $\;[{cm}^{3}]$	Power [W]	Multiple-Device	Radiated Magnetic Field	VFD $[{cm}^{-3}\cdot {Hz}^{-1}$]
Rotating magnet	[14]	75	>30	-	Yes	1 pT at 2771 m	≪4.44 × 10^−4^
	[13]	100–500	3	138	No	0.794 pT at 100 m	≪3.33 × 10^−3^
	[28]	1–5	125	-	No	20 nT at 5 m	≪8 × 10^−3^
Electret type	[18]	22.5	500	<5	No	5.63 nT at 8 cm	≪8.88 × 10^−5^
	[15]	100	16,000	-	No	100 fT at 92 m	6.25 × 10^−7^
	[29]	75	1000	-	No	117 pT at 100 m	≪1.33 × 10^−5^
Piezoelectric	[30]	33,600	≈50	1.2	No	0.23 fT at 1 km	5.95 × 10^−7^
	[31]	37,950	8.84	2.03	No	160 nT at 9 m	2.98 × 10^−6^
	[32]	1754	96,000	-	No	-	5.96 × 10^−9^
	[33]	91,800	0.17	-	No	1 pT at 3.3 m	6.4 × 10^−5^
	This work	9.7	6.6	0.0502	Yes	144 pT at 20 m	1.56 × 10^−2^

## Data Availability

Data supporting reported results can be provided upon request.
